# Functional Responses of Three Insect Predators to *Plutella xylostella* Across Developmental Stages

**DOI:** 10.3390/insects17050490

**Published:** 2026-05-11

**Authors:** Guanghua Liu, Yilin Xiong, Yunbo Song, Yuling Liang, Yongyue Lu

**Affiliations:** 1 College of Agriculture and Biology, Zhongkai University of Agriculture and Engineering, Guangzhou 510225, China; yilinxiong937@gmail.com; 2 Shaoguan Huashi Innovational Research Institute for Modern Agriculture, Shaoguan 512100, China; 3 College of Plant Protection, South China Agricultural University, Guangzhou 510642, China; songyunbo-scau@stu.scau.edu.cn (Y.S.); liangyuling@stu.scau.edu.cn (Y.L.)

**Keywords:** predator ontogeny, prey-stage effects, stage-structured predation, comparative predation, biological control

## Abstract

The diamondback moth, *Plutella xylostella*, is a major pest of cruciferous crops, and predatory insects are important candidates for its biological control. However, predator performance may vary substantially across developmental stages, complicating stage-specific evaluation and cross-species comparison. Here, we quantified corrected prey consumption and functional response patterns of three insect predators, *Eocanthecona furcellata*, *Hierodula patellifera*, and *Paratenodera sinensis*, across predator developmental stages, prey stages, and prey densities under laboratory conditions. Functional response types were classified using logistic regression, and model parameters were estimated using nonlinear functional response models. Corrected prey consumption generally increased with prey density, but the strength of density-dependent differentiation varied among predator instars and prey stages, with clearer treatment separation typically observed in later instars and adults than in early instars. Across 37 predator–prey-stage combinations, Type II-like functional responses predominated in 29 combinations, whereas 6 were classified as Type III-like and 2 were ambiguous. Strict matched-subset analysis further showed that relative differences among predator species were stage-dependent, indicating that cross-species comparisons were sensitive to predator developmental stage and the degree of experimental matching. These results indicate that the three predators have stage-dependent potential for the biological control of *P. xylostella* and provide a laboratory-based basis for their future evaluation and application under field conditions.

## Simple Summary

The diamondback moth, *Plutella xylostella*, is one of the most destructive pests of cruciferous crops worldwide. Predatory insects may help control this pest, but their effectiveness can vary substantially across developmental stages. This stage dependence is important in field biological control because predator stage and release timing may influence pest suppression. In this study, we evaluated three predators, *Eocanthecona furcellata*, *Hierodula patellifera*, and *Paratenodera sinensis*, under laboratory conditions to examine how predator developmental stage, prey stage, and prey density influenced feeding performance. All three predators generally consumed more prey when more prey were available, but their performance varied markedly across developmental stages. In general, later immature stages and adults showed stronger and more consistent predation than earlier stages. Comparisons among predator species also depended strongly on whether equivalent developmental stages were compared. These findings show that the biological control value of these predators is stage-dependent and provide a basis for selecting appropriate predator stages and informing future semi-field and field evaluation.

## 1. Introduction

The diamondback moth, *Plutella xylostella* (Linnaeus) (Lepidoptera: Plutellidae), is one of the most destructive pests of cruciferous crops worldwide [1,2]. Its global distribution, rapid life cycle, high fecundity, and migratory behaviour enable recurrent outbreaks that cause substantial economic losses [2,3,4,5]. Annual global management costs and crop damage are estimated at 4–5 billion USD, making it one of the most economically important agricultural pests [2,6]. In intensive vegetable production systems, particularly in southern China where year-round cultivation is common, *P. xylostella* populations persist continuously and exert sustained pressure on *Brassica* crops such as flowering Chinese cabbage [3,4].

Long-term reliance on chemical insecticides has led to widespread resistance in *P. xylostella*, including resistance to *Bacillus thuringiensis* toxins and multiple synthetic insecticides [7,8,9,10]. Resistance mechanisms involve target-site modification, metabolic detoxification, and altered toxin binding, greatly reducing chemical control efficacy [9,11]. As a result, biologically based management strategies have become central to sustainable pest control programmes [12,13].

Predatory insects are important components of integrated pest management [14,15]. Among them, predators such as *Eocanthecona furcellata* (Wolff) (Hemiptera: Pentatomidae), *Paratenodera sinensis* (Saussure) (Mantodea: Mantidae), and *Hierodula patellifera* (Serville) (Mantodea: Mantidae) have been reported to attack lepidopteran larvae [16,17,18,19]. However, most studies have evaluated predation capacity at single predator or prey stages, often focusing exclusively on larval prey [20]. Few studies have systematically examined how predator ontogeny interacts with prey ontogeny to shape predation dynamics across the full life cycle of *P. xylostella*, including larval, pupal (cocooned and naked), and adult stages [20,21,22]. Because prey functional traits such as body size, mobility, and defensive behaviour can change across developmental stages, their vulnerability to predators may also vary accordingly [23,24].

Functional response analysis provides a mechanistic framework for quantifying predator–prey interactions. In Holling’s framework, Type II responses are hyperbolic, with decelerating intake rates caused by handling-time limitation, whereas Type III responses are sigmoidal and can, in principle, stabilize predator–prey dynamics by reducing per capita predation at low prey densities and intensifying it at intermediate densities [25,26,27,28,29]. From a biological control perspective, such sigmoidal responses are often viewed as desirable because they imply stronger regulation at low to moderate pest densities. However, empirical evidence for true Type III responses remains relatively scarce and is strongly affected by statistical methods and experimental design [30,31,32]. Moreover, functional response type is not fixed: it can vary with predator taxon and body size, predator ontogeny, prey life stage and antipredator traits, foraging mode, temperature, and the presence of alternative prey [26,33,34,35,36,37].

Ontogenetic shifts in predator morphology and behaviour can substantially alter the attack rate (*a*) and handling time (*Th*), thereby modifying maximum predation capacity (*1/Th*) [38,39,40,41]. Likewise, prey developmental transitions—for instance, the acquisition of structural defences such as cocoons or harder exoskeletons—can impose additional handling constraints and reduce vulnerability [40,42,43]. As a result, predator–prey-stage combinations are expected to generate heterogeneous functional response shapes and parameter values. Although comparative work across predators with contrasting feeding modes indicates that functional traits and hunting modes strongly influence functional responses, quantitative, stage-explicit comparisons of response types and parameter variation between piercing–sucking and chewing predators attacking the same prey system remain scarce [27,34,44,45,46]. The three predators selected in this study represent contrasting feeding modes and are relevant to agroecosystems in which *P. xylostella* occurs. *E. furcellata* is a piercing–sucking predator, whereas *P. sinensis* and *H. patellifera* are chewing predators. These species have been reported from crop habitats where lepidopteran prey occur and may overlap spatially and seasonally with *P. xylostella* populations, making them suitable for evaluating stage-specific predation potential in a biologically relevant comparative framework. In addition, differences in experimental prey-density ranges often complicate interspecific comparisons. Robust evaluation of biological control potential therefore requires density-standardized comparisons and model validation across multiple functional response frameworks. Previous methodological studies have shown that variation in prey-density ranges and model choice can influence parameter estimation and complicate comparisons across predator taxa, highlighting the importance of standardized experimental designs when evaluating functional responses [47,48,49].

The present study systematically evaluated the density-dependent predation patterns of three insect predators—*Eocanthecona furcellata* (Wolff) (Hemiptera: Pentatomidae)*, Paratenodera sinensis* (Saussure) (Mantodea: Mantidae), and *Hierodula patellifera* (Serville) (Mantodea: Mantidae)—because they represent contrasting feeding modes and predator functional traits relevant to the biological control of *P. xylostella*. *E. furcellata* is a piercing–sucking predatory pentatomid that attacks lepidopteran larvae and has been evaluated as a potential biological control agent against diamondback moth under greenhouse conditions. By contrast, *P. sinensis* and *H. patellifera* are chewing, sit-and-wait generalist mantids that can capture mobile insect prey in crop habitats. These predators are likely to encounter *P. xylostella* in cruciferous cropping systems because the pest occurs on brassicaceous vegetables and its immature stages are exposed on host plants during crop growth. Therefore, overlap in crop habitat and potential temporal co-occurrence during pest outbreaks provide an applied rationale for comparing their stage-specific predation performance.

Accordingly, this study evaluated the density-dependent predation patterns of these three predators across multiple developmental stages of *P. xylostella*. Functional response analyses commonly use logistic regression to classify response type and nonlinear models, such as Holling’s disc equation or Rogers’ random predator equation, to estimate functional response parameters [47,50,51,52]. Following this established framework, we classified functional response types using logistic regression and estimated functional response parameters using nonlinear models. To improve cross-species comparability under the asymmetric experimental design, we further restricted species-level comparisons to shared prey-density intervals and matched predator–prey-stage combinations. The objectives were to (1) quantify stage-specific variation in predation performance across predator and prey developmental stages; (2) determine how functional response patterns and model parameters vary among predator–prey-stage combinations; and (3) assess whether feeding-mode differences between piercing–sucking and chewing predators affect cross-species comparability. We hypothesized that (1) predation performance would increase with predator development, with later stages showing higher and more stable prey consumption; (2) prey vulnerability would differ among developmental stages because of variation in body size, mobility, and defensive capacity, particularly between cocooned and non-cocooned pupae; and (3) functional response patterns and parameter estimates would differ between piercing–sucking and chewing predators, making cross-species comparisons stage-dependent.

## 2. Materials and Methods

### 2.1. Insect Colonies

The diamondback moth, *Plutella xylostella* (Linnaeus) (Lepidoptera: Plutellidae), was obtained from a commercial supplier—Henan Keyun Biotechnology Co., Ltd. (Jiyuan, China)—and reared for laboratory experiments. Individuals used as prey in the experiments were from the *F_2_* laboratory generation. Larvae were maintained on fresh seedlings of flowering Chinese cabbage under controlled conditions (27 ± 1 °C; 75 ± 5% RH; 14L:10D photoperiod) [53].

The predatory stink bug *Eocanthecona furcellata* (Wolff) (Hemiptera: Pentatomidae) was obtained from a laboratory colony maintained at Shaoguan Huashi Agricultural Innovation Institute (Shaoguan, China). The colony was reared on *Tenebrio molitor* pupae under 27 ± 1 °C, 75 ± 5% RH, and 16L:8D photoperiod, and *F_2_* individuals from this colony were used in the experiments [54].

Egg cases of the mantids *Paratenodera sinensis* (Saussure) (Mantodea: Mantidae) and *Hierodula patellifera* (Serville) (Mantodea: Mantidae) were collected from the Yanshan Mountains (Hebei Province, China) in November 2024. After hatching, mantids were reared individually and fed *Drosophila melanogaster* (Diptera: Drosophilidae) and *T*. *molitor* larvae according to instar requirements. Individuals used in the experiments were from the *F_1_* laboratory generation. Rearing conditions were 27 ± 1 °C, 70 ± 5% RH, and 14L:10D photoperiod. Because most predators used in this study were immature, reliable pre-assay sex determination was not feasible across all predator instars; therefore, predator sex was not experimentally controlled, and individuals were randomly selected for the predation assays.

Differences in laboratory generation among predator taxa reflected their source populations and should be considered when interpreting cross-species comparisons.

All experiments were conducted under controlled environmental conditions (27 ± 1 °C; 70–75% RH; 14L:10D). These conditions were selected with reference to published laboratory rearing protocols for the prey and predator species in order to maintain stable colony development and standardize comparisons under controlled conditions. They were not intended to fully simulate field environments [48,49]. Therefore, the results should be interpreted as laboratory-based estimates of predation performance rather than direct measures of field predation rates.

### 2.2. Rearing of P. xylostella

Larvae were maintained on potted flowering Chinese cabbage seedlings in climate-controlled chambers. Adults were allowed to oviposit on green paper strips treated with cabbage extract. Eggs were collected daily and surface-sterilized prior to use. Newly hatched larvae were transferred to fresh host plants and reared until the required developmental stage. Rearing procedures were based on standard laboratory methods for *P. xylostella* colony maintenance, with modifications for the purposes of stage-specific predation experiments [53].

### 2.3. Functional Response Experiments

In all assays, prey were placed directly into the experimental arena at the beginning of each trial, and a single predator was then introduced. Depending on the prey stage and assay type, a moist sponge, host plant material, or a honey-soaked sponge was provided to maintain prey survival and reduce artefactual mortality unrelated to predation. Behavioural interactions within the arenas were not systematically quantified, as the study focused on prey consumption outcomes and functional response estimation rather than detailed behavioural observation. Therefore, potential effects of arena structure and prey placement on predator searching behaviour should be considered when interpreting the results.

#### 2.3.1. Predation by *E. furcellata* on Larval Stages

Second-to-fifth-instar nymphs and adults of *E. furcellata* were tested. First-instar nymphs were excluded because previous laboratory observations of predatory pentatomids, including *E. furcellata*, indicate that newly hatched first instars may show limited or inconsistent prey-feeding behaviour and are less comparable to later stages in standardized predation assays [55,56,57,58]. Predators were fed *T. molitor* pupae for 24 h and then starved for 24 h prior to experimentation. Single predators were introduced into plastic Petri dishes (90 mm diameter; 15 mm height) containing different densities of *P. xylostella* larvae. A piece of moist sponge was placed in each dish to provide water for the predator. Fresh flowering Chinese cabbage seedlings or cut leaf pieces were also added to reduce cannibalism among *P. xylostella* larvae during the assay. Prey were grouped into three developmental categories: early instars (1st–2nd), 3rd instars, and 4th instars. Prey-density gradients were 5, 10, 15, 20, 30, and 40 individuals for 2nd–4th instar predators, and 10, 20, 40, 60, 80 and 100 individuals for 5th instars and adults. Each treatment was replicated five times. A no-predator control was included for each density to correct for natural mortality. After 24 h, surviving prey were counted and corrected consumption (*N_a_*) was calculated. General assay procedures followed standard laboratory functional response approaches, with modifications in prey-stage grouping, arena provisioning, and density design for the present study [47,59,60,61,62].

#### 2.3.2. Predation on Pupal Stages

Experiments were conducted using cocooned and manually de-cocooned pupae. The experimental design followed the same protocol as larval assays, including predator stages, density gradients, starvation regime, and replication. A piece of moist sponge was placed in each dish to provide water for the predator. Following 24 h exposure, predators were removed and pupae were maintained for 3–4 days to assess successful adult emergence. Consumption was corrected using control treatments.

#### 2.3.3. Predation by Mantids on Late-Instar Larvae

For both *T. sinensis* and *H. patellifera*, three predator stages were tested: early instars (1st–3rd), late instars (4th–7th), and adults. Starvation periods were predator-specific rather than standardized across taxa: 24 h for *E. furcellata* and 48 h for the mantids. These starvation periods followed laboratory preconditioning practices intended to promote active foraging during the assay; however, because starvation duration may influence both feeding motivation and physiological stress, this difference should be considered a potential limitation when interpreting direct cross-species comparisons. Each individual was used only once. Single mantids were placed in plastic containers (100 mL for early instars; 500 mL for later instars and adults) with 3rd–4th instar larvae of *P. xylostella*. Fresh flowering Chinese cabbage leaves were placed in each container to reduce cannibalism among *P. xylostella* larvae during the assay. Prey densities were 10, 20, and 30 individuals per container. Each treatment was replicated five times. Remaining prey were counted after 24 h.

#### 2.3.4. Predation on Adult Moths

The experimental design mirrored larval assays. Single mantids were exposed to adult *P. xylostella* at densities of 10, 20, and 30 individuals per container. A honey-soaked sponge was provided in each container to maintain prey survival during the assay and in the control treatments. Each treatment was replicated five times. Surviving moths were counted after 24 h.

## 3. Statistical Analyses

All statistical analyses were performed in R 4.4.0 [63]. Raw predation data were imported and standardized prior to modelling. Predator identity, predator life stage, prey stage, initial prey density (*N*), and corrected prey consumption (*N_a_*) were retained for analysis. *N* observations were removed as statistical outliers unless measurement error could be independently verified; no such cases were identified. All experimental replicates were treated as independent observations based on experimental design.

### 3.1. Density-Dependent Comparisons Within Predator–Prey Combinations

For descriptive comparisons of corrected prey consumption across prey densities within a given predator stage—prey-stage combination, nonparametric analyses were conducted using Kruskal–Wallis tests followed by Dunn’s multiple comparisons with Bonferroni adjustment where appropriate [64,65]. These analyses were used only to summarize within-combination density effects and were not used for cross-stage or cross-species inference. Because prey consumption data were not assumed to be normally distributed, nonparametric tests were preferred for these descriptive comparisons. Independence of observations was ensured by design, and the Kruskal–Wallis framework was chosen because it does not require normality. However, as with other rank-based methods, it assumes independent sampling and similarly shaped distributions across groups; results were therefore interpreted as evidence of distributional differences among density treatments rather than as strict differences in means.

### 3.2. Functional Response Classification

Functional response type was determined using logistic regression following Juliano (2001) [47]. For each predator–prey-stage combination, the proportion of prey consumed was modelled as follows:

$$\frac{N_{a}}{N_{0}}=\frac{\exp\left(P_{0}+P_{1}N_{0}+P_{2}N_{0}^{2}+P_{3}N_{0}^{3}\right)}{1+\exp\left(P_{0}+P_{1}N_{0}+P_{2}N_{0}^{2}+P_{3}N_{0}^{3}\right)}$$

where *P*_0_ is the intercept and *P*_1_*, P*_2_, and *P*_3_ are the linear, quadratic, and cubic coefficients, respectively. Higher-order terms were included to allow for flexible prey-dependent curvature within the observed density range [47].

Response type was interpreted primarily from the sign and significance of the first-order term: a significantly negative linear term was taken to indicate a Type II-like response, whereas a significantly positive linear term followed by a negative quadratic term was taken to indicate a Type III-like response [47]. However, logistic regression was used here as an initial classification aid rather than as a definitive test of the underlying mechanistic response form. This distinction is important because empirical identification of functional response shape can be influenced by prey-density range, sample size, model specification, and experimental design, and because over-reliance on Type II/III classification may overstate the certainty of response-form inference [31,32].

Alternative response forms were considered conceptually but were not treated as primary candidates for formal classification in the present study. A true Type I response was not regarded as biologically plausible because all three predator species necessarily exhibit non-zero handling time. Dome-shaped or Type IV responses were also considered, as such forms are recognized in the broader functional response literature, but they were not inferred from the present dataset because prey consumption did not decline at the highest prey densities tested. Accordingly, formal classification in this study was restricted to Type II-like versus Type III-like patterns within the experimental range, while acknowledging that other forms cannot be excluded outside that range [31,66].

Because corrected consumption values occasionally yielded non-integer responses, the logistic models were treated as proportion-based classification tools rather than strict binomial count models. Final interpretation of response type was therefore based jointly on logistic classification, biological plausibility, and comparative model fitting, rather than on Type II/III labels alone [32,66].

### 3.3. Functional Response Parameter Estimation

Functional response parameters were estimated using Holling’s Type II model (Holling, 1959) [50]:

$$N_{a}=\frac{aN_{0}}{1+\mathrm{aTh}N_{0}}$$

where *a* represents attack rate and *Th* denotes handling time. Parameter estimation was conducted using constrained nonlinear least squares (nlsLM), with biologically realistic parameter bounds (*a* ≥ 0; *Th* ≥ 0). Maximum predation capacity was calculated as *1/Th*.

Because parameter estimates may depend on model structure and data support, Holling’s Type II model was treated as the primary analytical representation only when supported by overall fit and biological plausibility. Model convergence, boundary behaviour, and residual patterns were inspected to evaluate numerical stability and to identify cases in which parameter estimates were weakly identified or approached limiting solutions. Functional response parameter inference and the biological interpretation of handling time are known to be sensitive to experimental design and statistical treatment, and were therefore interpreted cautiously [52].

### 3.4. Model Robustness Evaluation

To evaluate numerical stability and alternative model fit, three complementary modelling frameworks were considered: Holling’s Type II model, Rogers’ random predator equation for non-replacement designs, and generalized additive models (GAMs) as flexible nonparametric trend references. Candidate models were compared using Akaike’s Information Criterion (AIC). Rogers’ equation was included because it explicitly accounts for prey depletion under non-replacement conditions [51], GAMs were included to describe empirical curvature without imposing a fixed mechanistic shape [67], and AIC was used as a standard criterion for relative model comparison [68].

This multi-model strategy was adopted because classification based solely on polynomial logistic regression may be unstable when prey-density coverage is limited or when alternative curve shapes are weakly separated. The purpose of robustness analysis was therefore not only to improve numerical fit, but also to reduce the risk of drawing qualitative conclusions from a single classification method [31,32].

### 3.5. Strict Cross-Species Comparison Under Matched Conditions

Because predator stages, prey stages, and prey-density ranges were not fully aligned among predator species, direct cross-species comparisons were restricted to the strict matched subset consisting of the shared prey stage (4th instar), shared predator stages (2nd and 3rd instars), and shared prey densities (*N* = 10, 20, and 30). Within this matched subset, comparative analyses were based on corrected prey consumption (*N_a_*) and predation efficiency rather than on re-estimated functional response parameters, in order to minimize design-induced bias and avoid overinterpretation of parameter differences outside comparable experimental contexts.

Predation efficiency was quantified as

$$\mathrm{Efficiency}=\frac{Na}{N}$$

and was treated as a descriptive, model-independent index of realized predation performance. Corrected prey consumption was analyzed using linear models, whereas efficiency was analyzed using quasibinomial or beta-regression-based frameworks as appropriate to the response distribution.

For linear models, residual plots were inspected to assess gross deviations from homoscedasticity, normality, and overall model fit. Outliers were inspected graphically and by residual diagnostics. No observations were removed unless they reflected clear data-entry or procedural errors. These models were used here for comparative description within the matched subset rather than for extrapolative prediction. For beta regression, the response variable must lie within the open interval (0,1). Therefore, observations equal to 0 or 1 were excluded only from beta-regression fitting, because such boundary values violate the distributional assumption of the beta family and can otherwise lead to distorted parameter estimation.

To avoid inference depending on this exclusion step, all boundary observations were retained in complementary quasibinomial analyses, which are valid for proportions including 0 and 1. Thus, beta regression was used as a distribution-sensitive secondary framework for interior proportion values, whereas the quasibinomial model served as the primary robustness check for the full dataset. In this way, exclusion of boundary values affected only model fitting for beta regression, not the overall biological interpretation. Pairwise comparisons were performed using Tukey-adjusted estimated marginal means.

## 4. Results

### 4.1. Density-Dependent Variation in Corrected Predation by E. furcellata

**Figure 1:**
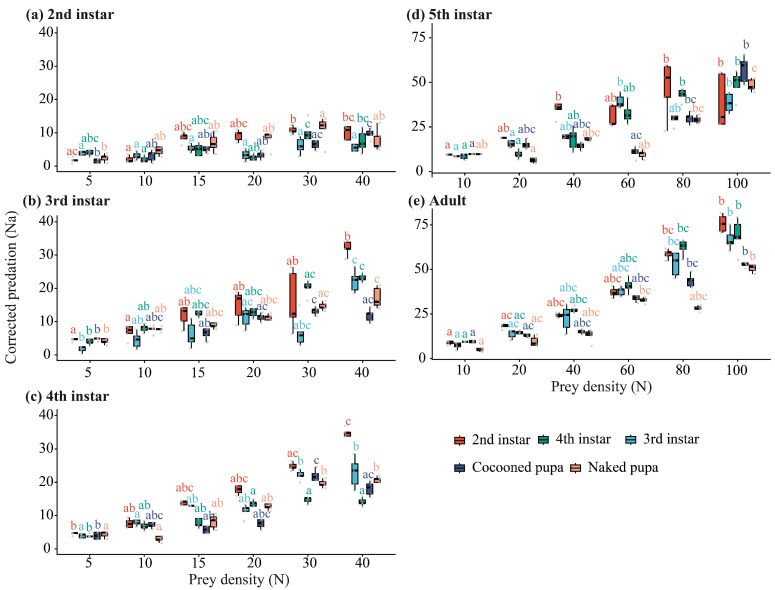
Density-dependent variation in corrected predation (*N_a_*) of *E. furcellata* across predator developmental stages. Corrected predation (*N_a_*) of *E. furcellata* on different developmental stages of *P. xylostella* under different initial prey densities. Panels show predator stages: (**a**) 2nd instar, (**b**) 3rd instar, (**c**) 4th instar, (**d**) 5th instar, and (**e**) adult. Colours indicate prey stages of *P. xylostella*. Boxes show the interquartile range, the horizontal line within each box indicates the median, whiskers represent 1.5 × IQR, and points represent individual observations. Different lowercase letters indicate significant differences among prey-density treatments within the same prey stage (Kruskal–Wallis tests followed by Dunn’s multiple comparisons, *p* < 0.05).

Prey density significantly affected corrected predation (*N_a_*) by *E. furcellata* in all predator–prey-stage combinations tested (Figure 1). Corrected predation generally increased with prey density, but the strength of density-dependent differentiation varied across predator and prey stages.

For 2nd–4th instar predators, tested at prey densities of 5, 10, 15, 20, 30, and 40 individuals, all overall density effects were significant (all *p* ≤ 0.013 for 2nd instars; all *p* < 0.001 for 3rd–4th instars). Corrected predation was generally lowest at the lowest prey densities and increased toward the upper density treatments. For example, in 2nd-instar predators feeding on 2nd-instar prey, mean *N_a_* increased from 1.73 ± 0.32 at density 5 to 10.78 ± 0.51 at density 30.

For 5th-instar predators and adults, tested at prey densities of 10, 20, 40, 60, 80, and 100 individuals, density effects remained significant across all prey-stage combinations (all *p* < 0.001). Later predator stages generally showed higher corrected predation and clearer density-dependent differentiation than earlier stages. In adults feeding on 2nd-instar prey, mean *N_a_* increased from 8.86 ± 0.40 at density 10 to 75.81 ± 2.15 at density 100.

Overall, *E. furcellata* showed a progressive density-dependent increase in corrected predation rather than complete pairwise separation across all density levels. The clearer response in later predator stages suggests that predation capacity and density responsiveness strengthened with predator development.

### 4.2. Density-Dependent Predation Patterns of H. patellifera and P. sinensis

**Figure 2:**
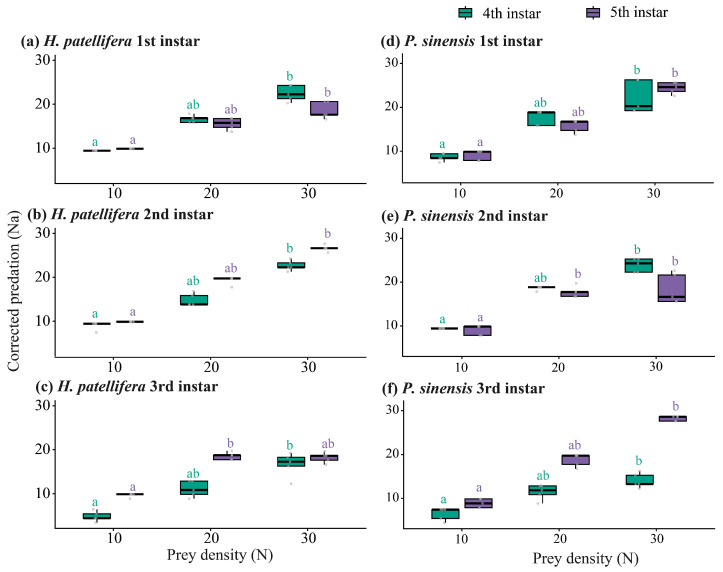
Density-dependent variation in corrected predation (*N_a_*) by *H. patellifera* and *P. sinensis* across predator developmental stages. Corrected predation (*N_a_*) of *H. patellifera* and *P. sinensis* on 4th- and 5th-instar *P. xylostella* larvae under different initial prey densities (*N*). Different colours indicate prey stages. Boxes represent the interquartile range, centre lines indicate medians, whiskers extend to 1.5 × IQR, and points represent individual observations. Different lowercase letters indicate significant differences among prey-density treatments within the same prey stage (Kruskal–Wallis tests followed by Dunn’s multiple comparisons, *p* < 0.05).

Prey density significantly affected corrected predation (Na) in all tested predator–prey-stage combinations of both *H. patellifera* and *P. sinensis* (Figure 2). In both mantids, corrected predation increased with prey density, but pairwise separation was generally clearer between the lowest and highest densities than between adjacent density levels.

In *H. patellifera* (Figure 2a–c), all three predator instars showed significant density dependence when feeding on both 4th- and 5th-instar prey. For example, in 1st-instar predators, mean *N_a_* increased from 9.42 ± 0.00 to 22.47 ± 0.80 for 4th-instar prey and from 9.87 ± 0.00 to 18.62 ± 0.84 for 5th-instar prey between densities 10 and 30.

A comparable pattern was observed in *P. sinensis* (Figure 2e,f). In 1st-instar predators, mean *N_a_* increased from 8.62 ± 0.37 to 22.27 ± 1.64 for 4th-instar prey and from 9.07 ± 0.49 to 24.42 ± 0.58 for 5th-instar prey between densities 10 and 30.

Taken together, both mantid predators showed positive density-dependent prey consumption, but the response was gradual rather than sharply threshold-like. This indicates that higher prey availability increased realized consumption, while the lack of full separation among adjacent densities suggests continuous rather than discrete density-dependent changes.

### 4.3. Functional Response Patterns and Parameter Estimates

**Figure 3:**
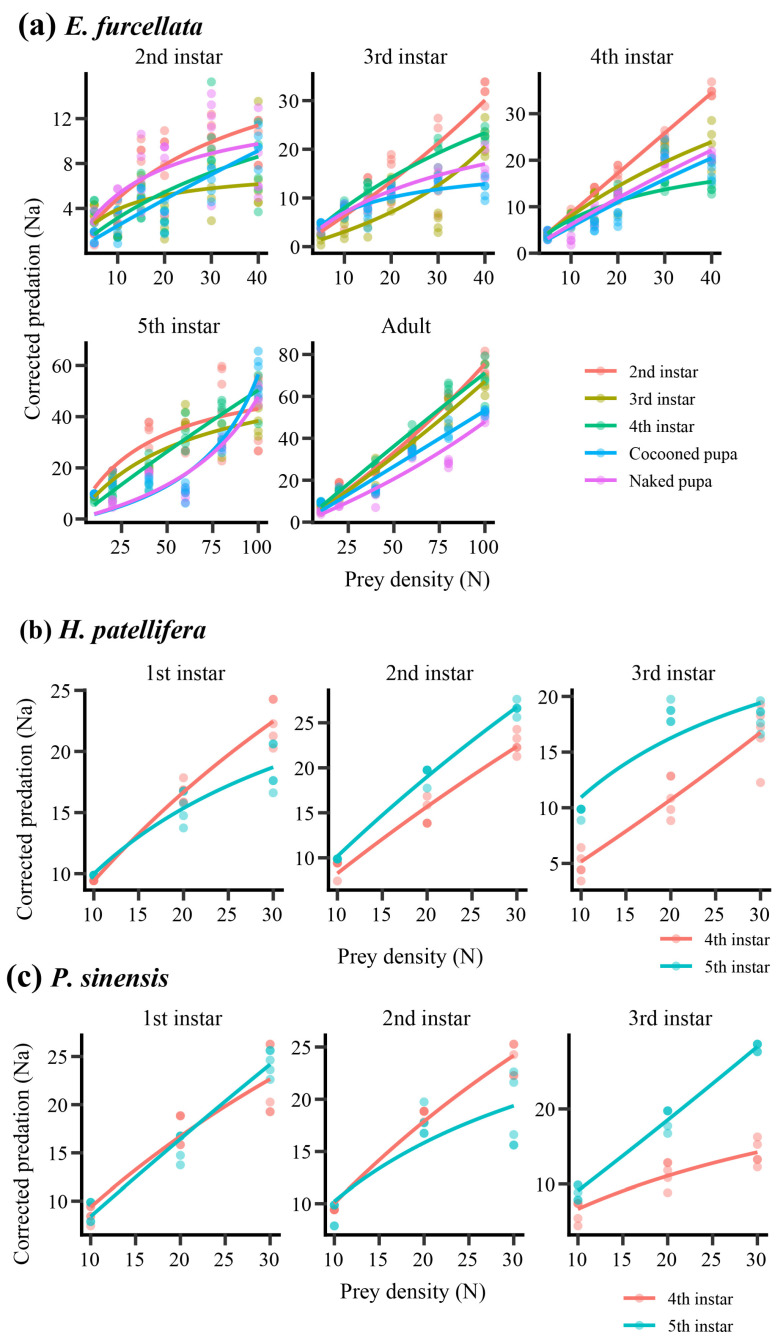
Functional response curves across predator and prey developmental stages. Observed and fitted functional response relationships between prey density (*N*) and corrected predation (*N_a_*) for *E. furcellata* (**a**), *H. patellifera* (**b**), and *P. sinensis* (**c**). *N* indicates the number of prey initially provided, and *Na* indicates corrected prey consumption after adjustment for prey mortality in the no-predator control. Within each species, subpanels correspond to predator developmental stages, and line colours denote prey developmental stages. Points represent observed mean values, and solid lines represent fitted functional response curves based on the primary model recommended for each predator–prey-stage combination, either Holling’s Type II equation or Rogers’ random predator equation. The model used for each fitted curve is listed in Table S1.

Functional response curves generally increased with prey density across predator–prey combinations, although the inferred response form varied among predator species and developmental-stage combinations (Figure 3). Across the 37 predator stage–prey-stage combinations examined, Type II-like responses predominated (29 combinations), whereas six combinations were classified as Type III-like and two were classified as ambiguous (Table 1). The predominance of Type II-like responses was largely driven by *E. furcellata*, for which 22 of 25 combinations exhibited decelerating intake rates at higher prey densities. In contrast, *P. sinensis* showed greater heterogeneity in response curvature, including three Type III-like responses, suggesting potential variation in prey-stage-specific attack behaviour or prey handling constraints.

Model recommendation varied among predator–prey combinations. Holling’s Type II model was selected as the primary fitted model in 21 combinations, whereas Rogers’ random predator equation was preferred in 16 combinations (Table 1). Rogers’ model was more frequently selected for *P. sinensis*, reflecting the increased likelihood of prey depletion during experimental trials. Model recommendations indicate relative statistical support within each predator–prey combination but should not be interpreted as direct evidence for mechanistic differences among predator species.

Detailed parameter estimates are provided in Supplementary Table S1. Handling-time estimates (*Th)* were frequently weakly identified, reflecting limited information content in the observed intake curves at high prey densities. Of the 37 combinations, only one yielded a stable *Th* estimate, whereas 21 were classified as weakly identified, 13 as boundary estimates, and two as non-identifiable (Table 1). Consequently, maximum predation capacity (*1/Th*) was considered directly interpretable in only one combination. Overall, these results indicate that Type II-like response curvature predominated across predator species, although parameter identifiability varied substantially among predator–prey-stage combinations.

### 4.4. Strict Cross-Species Comparison Within the Matched Subset

**Figure 4:**
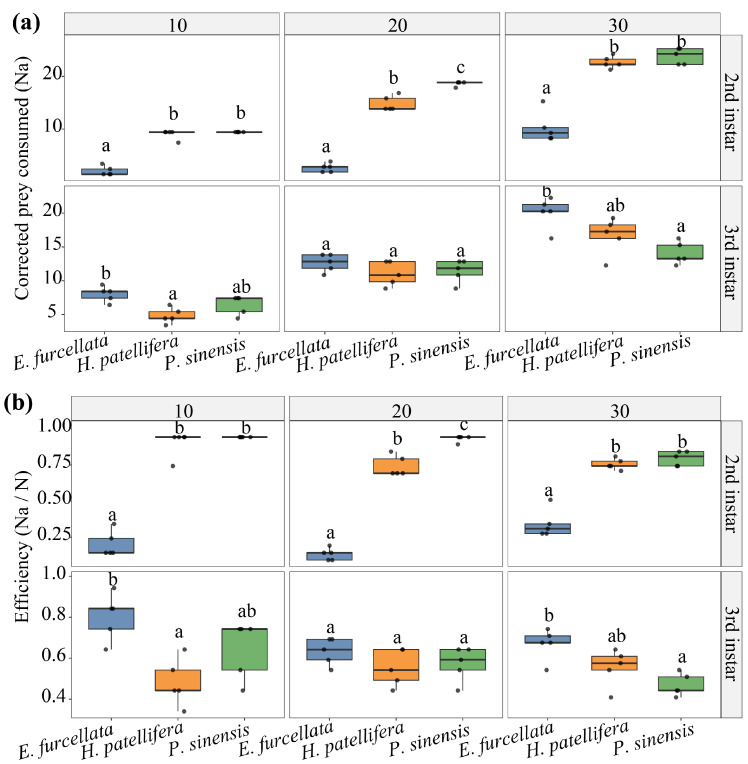
Strict cross-species matched comparisons on 4th-instar prey. Only the shared predator stages (2nd and 3rd instars) and shared prey densities (10, 20, and 30) are shown. Panel (**a**) presents corrected prey consumption (*N_a_*), and panel (**b**) presents predation efficiency (*N_a_*/*N*), for *E. furcellata*, *H. patellifera*, and *P. sinensis*. Columns indicate prey density, and rows indicate predator stage. Different lowercase letters denote Tukey-adjusted within-cell groupings. These boxplots are descriptive summaries of the strict matched subset and do not replace functional response curve analysis.

Cross-species comparison was restricted to a partially matched subset defined by the shared prey stage (4th instar), shared predator stages (2nd and 3rd instars), and shared prey densities (10, 20, and 30) (Figure 4a). Because starvation time and arena differed among predator taxa, these comparisons were limited to laboratory prey-removal patterns under partially matched conditions. For corrected prey consumption, the full interaction model was strongly preferred over an additive model (AIC = 353.97 vs. 514.61), indicating that differences among predator species depended on predator stage and prey density.

Pairwise contrasts supported a stage-dependent reversal in relative performance (Figure 4b). In 2nd instars, *E. furcellata* showed significantly lower corrected prey consumption than both mantid species at all three shared densities (all adjusted *p* ≤ 0.01). In 3rd instars, this pattern no longer held consistently: *E. furcellata* exceeded *H. patellifera* at *N* = 10 and 30, exceeded *P. sinensis* at *N* = 30, and did not differ significantly from either mantid in several remaining contrasts. Corrected efficiency (*N_a_*/*N*) showed the same qualitative stage dependence, with *E. furcellata* lower than both mantids in 2nd instars but not consistently lower in 3rd instars. Raw and corrected analyses yielded the same directional conclusions across the matched-subset contrasts.

## 5. Discussion

The present study shows that predation on *P. xylostella* was strongly density-dependent across the three predator species examined, but the magnitude and form of this response varied with predator ontogeny and prey developmental stage. Across the full dataset, corrected prey consumption generally increased with prey density, and Type II-like functional responses predominated. However, response form was not uniform across all predator stage and prey-stage combinations, and parameter identifiability—particularly for handling time—was often limited. These results indicate that predator stage, prey stage, and predator identity jointly shape predation performance, but also that some parameter-based comparisons should be interpreted cautiously.

### 5.1. Ontogenetic Changes in Predation Capacity

Predation capacity generally increased with predator development, supporting our first hypothesis. In *E. furcellata*, later instars and adults exhibited markedly higher prey consumption compared with early instars. Similar ontogenetic increases in predation efficiency have been widely documented in predatory insects and are typically associated with increases in body size, mobility, and feeding capability [16,57,69,70]. Larger predators are able to subdue prey more rapidly and process prey more efficiently, thereby increasing both attack rate and maximum predation capacity.

Early instars of *E. furcellata* showed relatively limited predation ability, particularly when attacking larger prey stages. This likely reflects morphological and behavioural constraints during early development, including limited rostrum strength and lower locomotor performance. As predator size increases, these constraints are progressively reduced, enabling later instars to exploit a wider range of prey sizes. Similar ontogenetic expansions in prey utilization have been reported for other predatory hemipterans and generalist arthropod predators [71,72].

### 5.2. Influence of Prey Developmental Stage

Prey developmental stage strongly influenced predation dynamics, particularly in *E. furcellata*. Handling time varied among prey stages, indicating that prey morphology and defensive traits impose different constraints on predator foraging efficiency. Larval prey generally required longer handling times, likely due to their higher mobility and active defensive behaviours. In contrast, pupal prey were immobile and therefore easier to capture, which likely explains the reduced handling times and elevated maximum predation capacities observed for these prey stages.

Interestingly, the presence of cocoon silk around pupae did not prevent predation by *E. furcellata*, although it may impose additional handling constraints. Structural defences such as cocoons or thicker cuticles are known to alter predator–prey interactions by increasing manipulation time or reducing capture success [67,68,69]. The ability of *E. furcellata* to successfully attack both cocooned and naked pupae suggests a relatively flexible feeding strategy that may allow for exploitation of multiple prey stages in field conditions.

### 5.3. Functional Response Patterns and Predator Feeding Strategies

Functional response analyses revealed that Type II responses predominated across most predator–prey combinations, although Type III responses occurred in several stage combinations. The predominance of Type II-like responses is consistent with many studies of arthropod predators, in which prey intake tends to become constrained by handling time as prey density increases [38,69,73]. Under a Type II response, per capita predation rate typically increases rapidly at low prey densities but gradually saturates as handling constraints accumulate, reflecting a decelerating increase in prey consumption with increasing prey availability.

Type III responses, although less frequent, were observed in several predator–prey-stage combinations, particularly in mantid predators and early predator stages. Sigmoidal responses are often associated with prey switching, learning behaviour, or density-dependent increases in predator search efficiency [31]. One possible explanation is that predator stages with limited initial capture efficiency may improve their ability to locate, subdue, or process prey after repeated encounters, resulting in accelerated prey consumption once prey density exceeds a threshold level. Such density-dependent improvements in prey exploitation efficiency may generate the characteristic sigmoid relationship between prey density and predation rate.

However, the ecological interpretation of Type III responses remains debated. Apparent sigmoidal functional responses may sometimes arise as statistical artefacts caused by limited prey-density ranges, insufficient replication, or model-selection procedures, rather than representing stable predator behavioural strategies [32]. This concern is particularly relevant when prey-density gradients are sparse or uneven across predator–prey combinations, because apparent response-form classification can be sensitive to density range, replication, and model specification [31,32].

Functional response patterns estimated under simplified laboratory conditions may also differ from realized predator performance in field environments. In natural crop systems, plant architecture, spatial refuges, and habitat complexity can reduce encounter probability between predators and prey and thereby alter density dependence relative to laboratory estimates [66]. In addition, interactions among multiple predators, including predator interference and intraguild predation, may further modify realized prey consumption in multi-enemy systems [74]. Therefore, laboratory-derived functional response types should be viewed as mechanistic approximations that help explain predator–prey interaction processes but may not directly predict pest suppression strength under structurally complex field conditions.

### 5.4. Comparative Predation Efficiency Among Predator Species

Clear interspecific differences in predation efficiency were detected among the three predators examined. Both mantid species, *P. sinensis* and *H. patellifera*, exhibited significantly higher predation efficiencies than *E. furcellata*, indicating substantial variation in prey exploitation capacity among predator taxa.

These interspecific differences likely reflect contrasts in feeding mode and foraging behaviour. Mantids are raptorial predators that capture prey using specialized forelegs and consume prey through chewing mouthparts, allowing for rapid prey capture and relatively short handling times. This feeding strategy facilitates high instantaneous consumption rates when prey are abundant. In contrast, *E. furcellata* is a piercing–sucking predator that immobilizes prey using a rostrum and consumes liquefied tissues. Such feeding behaviour generally requires longer prey-processing time and may constrain maximum intake rates, particularly at high prey densities where handling becomes a limiting factor [36,75].

Differences in feeding mode may also reflect contrasting constraints associated with prey capture and processing. Raptorial predators rely on rapid strike movements and mechanical prey fragmentation, which may allow for rapid prey utilization when encounter rates are high. In contrast, piercing–sucking predators typically require extended feeding periods for tissue extraction and ingestion, potentially resulting in more gradual changes in realized consumption across prey-density ranges. Such differences in prey-processing constraints may influence predator performance under fluctuating prey densities, as predators limited primarily by handling or digestion processes may respond differently to temporal variation in prey availability [62,63,64]. From an optimal-foraging perspective, these feeding strategies may also involve different time-allocation and energetic trade-offs, because prey profitability depends jointly on capture effort, handling time, and energetic gain [76,77].

These results are consistent with comparative studies indicating that predator functional traits, including feeding mode, hunting strategy, and body size, strongly influence functional response parameters and predation efficiency [75,78,79]. Trait-mediated differences in predator behaviour can modify prey consumption rates and shape predator–prey interaction strength, ultimately influencing the effectiveness and stability of biological control systems [80].

### 5.5. Implications for Biological Control of P. xylostella

From an applied perspective, the results suggest that different predator taxa may play complementary roles in the biological control of *P. xylostella*. Mantid predators exhibited higher per capita predation rates, suggesting strong potential for suppressing dense pest populations. However, practical biological-control value depends not only on per capita predation, but also on mass-rearing feasibility, post-release persistence, dispersal ability, and compatibility with other natural enemies and control tactics. In augmentative biological control, realized field performance is often shaped by post-release survival, habitat structure, and antagonistic interactions such as predator interference or intraguild predation rather than by laboratory feeding rates alone [66,74,81].

In contrast, *E. furcellata* demonstrated the ability to exploit multiple prey developmental stages, including larvae and pupae. Although its per capita predation rate was lower than that of mantids, its smaller body size, higher population density, and ability to persist within crop habitats may enhance its practical value as a biological control agent [82]. The capacity to attack pupae may be particularly important because this stage can be less accessible to other natural enemies and may escape effective suppression when control relies mainly on larval-stage targeting.

These findings therefore support a complementary rather than single-agent view of biological control. Predator taxa with different prey-stage preferences and functional traits may contribute differently across pest-density contexts. In particular, mantid predators may be more effective for rapid suppression of abundant larval prey under high-density conditions, whereas *E. furcellata* may provide complementary value because of its broader prey-stage use and the existence of mass-rearing and applied biocontrol studies for this predator [82,83]. Their applied value is therefore likely to be greatest when integrated with other tactics already used in diamondback moth management, including parasitoids and microbial insecticides such as *Bacillus thuringiensis* [84]. At the same time, the extent to which such complementarity can be realized in practice will depend on field survivorship, dispersal, and interactions among natural enemies, because predator interference and intraguild predation can alter realized pest suppression in multi-enemy systems [74]. Accordingly, laboratory functional response results are most informative when interpreted as part of a broader integrated pest management framework rather than as direct evidence that any one predator alone would provide superior field suppression.

### 5.6. Limitations and Future Research Directions

Although laboratory functional response experiments provide useful mechanistic insight into predator–prey interactions, several limitations should be considered when interpreting the present results and extrapolating them to biological control under field conditions. First, prey were introduced directly into confined laboratory arenas, and behavioural interactions were not formally recorded. Under these simplified conditions, potential effects of arena structure on predator searching behaviour, prey escape responses, and encounter frequency could not be evaluated. More broadly, environmental complexity, plant architecture, prey refuges, and the availability of alternative prey may substantially modify predator behaviour, encounter rates, and functional response parameters under natural conditions [85].

A second limitation concerns the comparability of predator taxa under the present assay design. Starvation preconditioning was not standardized across taxa (24 h for *E. furcellata* versus 48 h for the mantids). Although these predator-specific starvation periods were selected to promote active foraging, variation in starvation duration may alter feeding motivation, prey acceptance, and physiological state, thereby affecting direct interspecific comparisons of predation performance and fitted functional response parameters. Accordingly, cross-species differences reported here should be interpreted as comparative patterns under the present experimental conditions rather than starvation-independent estimates of intrinsic predatory capacity.

A third limitation is the lack of direct behavioural evidence. Because predator attacks were not recorded in detail, behavioural components such as attack success, prey rejection, handling behaviour, prey escape, and search trajectories could not be quantified. Consequently, variation in attack rate and handling time could only be inferred indirectly from fitted functional response models rather than demonstrated through direct behavioural observation [52]. These parameters should therefore be interpreted as phenomenological descriptors of predation under the present assay conditions, rather than as direct measurements of the underlying behavioural mechanisms.

In addition, the present study did not include replicated semi-field or field validation. It therefore remains uncertain to what extent laboratory-derived predation rates and functional response parameters translate into realized suppression of *P. xylostella* under agronomic conditions, where predator survivorship, dispersal, habitat structure, and interactions with other natural enemies may strongly influence operational performance [66,81]. Interactions among multiple predator species, including predator interference and intraguild predation, may further modify pest suppression in multi-enemy systems [74]. Future studies should therefore assess predator performance under semi-field and field conditions, explicitly test the effects of habitat complexity, temperature variation, and alternative prey on predation dynamics, and standardize or experimentally manipulate starvation regimes across predator taxa. Integrating functional response analyses with numerical response or population-level models would also improve predictions of long-term biological control outcomes [52]. In addition, evaluating predator coexistence, compatibility with parasitoids and microbial control agents, and complementary predation among multiple natural enemies would provide a stronger basis for optimizing integrated management strategies against *P. xylostella* [84].

## 6. Conclusions

Predation by *E. furcellata*, *H. patellifera*, and *P. sinensis* on *P. xylostella* was strongly density-dependent, but highly contingent on predator developmental stage and prey stage. Type II-like functional responses predominated overall, although response patterns varied among predator taxa and ontogenetic stages. Cross-species differences were also stage-dependent and sensitive to experimental matching, indicating that predator performance should not be interpreted from stage-aggregated estimates alone. From an applied perspective, the results suggest that later instars of the mantid predators may be the most promising candidates for rapid suppression of high-density larval populations under laboratory conditions, owing to their comparatively high per capita predation rates. In contrast, *E. furcellata* may provide complementary value because of its broader prey-stage use, including the ability to attack pupae, a stage that may be less effectively suppressed by other natural enemies. These findings therefore support a stage-explicit and functionally complementary view of predator use in biological control rather than reliance on a single predator taxon or life stage.

However, these conclusions remain provisional until validated under replicated semi-field and field conditions. Future research integrating functional response with numerical response, behavioural observations, and population-level modelling will be essential for predicting which predator combinations are most likely to provide reliable suppression of *P. xylostella* in practice.

## Figures and Tables

**Table 1:** Functional response classification, preferred fitted model, and handling-time identifiability across predator species.

Predator Species	No. of Combinations	Type II-Like	Type III-Like	Ambiguous	Holling II Preferred	Rogers Preferred	Stable *Th*	Weakly Identified *Th*	Boundary Estimate	Non-Identifiable *Th*	Reportable *1/Th*
*E. furcellata*	25	22	3	0	15	10	1	12	10	2	1
*H. patellifera*	6	5	0	1	4	2	0	5	1	0	0
*P. sinensis*	6	2	3	1	2	4	0	4	2	0	0
Total	37	29	6	2	21	16	1	21	13	2	1

Notes: Numbers refer to predator–prey developmental-stage combinations. Functional response type was classified as Type II-like, Type III-like, or ambiguous according to the logistic-regression-based screening procedure described in the Methods. “Holling II preferred” and “Rogers preferred” indicate the number of combinations for which each model was selected as the primary fitted model based on relative model support. Handling-time identifiability was summarized using four categories. Stable *Th* indicates that the handling-time estimate converged with a biologically plausible value and was considered sufficiently reliable for interpretation. Weakly identified *Th* indicates that the model converged, but the estimate was poorly constrained, typically with limited precision or a relatively flat likelihood surface. Boundary estimate indicates that the estimate approached the optimization boundary, suggesting that *Th* could not be reliably resolved from the available data. Non-identifiable *Th* indicates that *Th* could not be estimated reliably because of model non-convergence or severe parameter instability. “Reportable *1/Th*” denotes combinations for which maximum predation capacity was considered sufficiently reliable for direct reporting.

## Data Availability

The original contributions presented in this study are included in the article/Supplementary Material. Further inquiries can be directed to the corresponding authors.

## Supplementary Materials

The following supporting information can be downloaded at: https://www.mdpi.com/article/10.3390/insects17050490/s1, Table S1. Functional response classification, fitted Holling Type II parameters, and reporting diagnostics for all predator–prey-stage combinations.
