# Supplementary material for: Functional Responses of Three Insect Predators to Plutella xylostella Across Developmental Stages
Source: Insects. 2026 May 11;17(5):490. doi: 10.3390/insects17050490 (PMC13207512; doi:10.3390/insects17050490)
Supplement: Supplementary file 1 [file insects-17-00490-s001.zip › insects-4276223-supplementary.pdf]

Table S1. Functional response classification, fitted Holling type II parameters, and reporting diagnostics for all predator - prey stage combinations

Predator species	Predator stage	Prey stage	n	Density levels	Design class.	Response form	Evidence	Attack rate (*a*)	Handling time (*Th*)	*Th* SE	Th CI	*Th* identifiability	*1/Th*	Holling II AIC	Primary model recommendation
E. furcellata	2nd instar	2nd instar	30	6	rich_density_design	Type III-like	moderate	0.635	0.0483	0.0128	[0.0248, 0.0711]	Weakly identified	NA	134.0	Rogers
E. furcellata	2nd instar	3rd instar	30	6	rich_density_design	Type II-like	moderate	0.803	0.131	0.0299		stable	7.614	134.8	HollingII
E. furcellata	2nd instar	4th instar	30	6	rich_density_design	Type II-like	strong	0.37	0.0486	0.0337		Weakly identified	NA	149.0	HollingII
E. furcellata	2nd instar	Cocooned pupa	30	6	rich_density_design	Type II-like	moderate	0.252	0.0101	0.0216		Non identifiable	NA	110.5	HollingII
E. furcellata	2nd instar	Naked pupa	30	6	rich_density_design	Type II-like	moderate	0.852	0.0733	0.018	[0.0396, 0.108]	Weakly identified	NA	149.4	Rogers
E. furcellata	3rd instar	2nd instar	30	6	rich_density_design	Type III-like	moderate	0.718	0	0.0078		Boundary estimate	NA	181.9	HollingII
E. furcellata	3rd instar	3rd instar	30	6	rich_density_design	Type III-like	strong	0.45	0	0.0185		Boundary estimate	NA	177.9	HollingII
E. furcellata	3rd instar	4th instar	30	6	rich_density_design	Type II-like	moderate	0.903	0.0151	0.0027	[0.0095, 0.0205]	Weakly identified	NA	112.8	HollingII
E. furcellata	3rd instar	Cocooned pupa	30	6	rich_density_design	Type II-like	strong	1.183	0.0567	0.0067	[0.043, 0.0705]	Weakly identified	NA	124.3	HollingII
E. furcellata	3rd instar	Naked pupa	30	6	rich_density_design	Type II-like	moderate	0.89	0.0308	0.004	[0.0223, 0.0391]	Weakly identified	NA	109.9	HollingII
E. furcellata	4th instar	2nd instar	30	6	rich_density_design	Type II-like	moderate	0.861	0	0.0014		Boundary estimate	NA	99.91	HollingII
E. furcellata	4th instar	3rd instar	30	6	rich_density_design	Type II-like	moderate	0.899	0.014	0.0045	[0.0046, 0.0229]	Weakly identified	NA	145.9	Rogers
E. furcellata	4th instar	4th instar	30	6	rich_density_design	Type II-like	moderate	1.026	0.0406	0.0051	[0.0308, 0.0504]	Weakly identified	NA	120.1	Rogers
E. furcellata	4th instar	Cocooned pupa	30	6	rich_density_design	Type II-like	strong	0.589	0.0064	0.0102		Weakly identified	NA	164.4	Rogers
E. furcellata	4th instar	Naked pupa	30	6	rich_density_design	Type II-like	strong	0.639	0.006	0.0052		Weakly identified	NA	133.7	Rogers
E. furcellata	5th instar	2nd instar	30	6	rich_density_design	Type II-like	moderate	1.483	0.0165	0.0031	[0.0099, 0.0224]	Weakly identified	NA	223.9	Rogers
E. furcellata	5th instar	3rd instar	30	6	rich_density_design	Type II-like	moderate	1.005	0.0162	0.0027	[0.0104, 0.0213]	Weakly identified	NA	191.0	Rogers
E. furcellata	5th instar	4th instar	30	6	rich_density_design	Type II-like	strong	0.552	0.0017	0.0026		Non identifiable	NA	183.9	Rogers
E. furcellata	5th instar	Cocooned pupa	30	6	rich_density_design	Type II-like	strong	0.44	0	0.0076		Boundary estimate	NA	226.9	HollingII
E. furcellata	5th instar	Naked pupa	30	6	rich_density_design	Type II-like	strong	0.394	0	0.0072		Boundary estimate	NA	210.5	HollingII
E. furcellata	adult	2nd instar	30	6	rich_density_design	Type II-like	strong	0.721	0	0.0013		Boundary estimate	NA	179.6	HollingII
E. furcellata	adult	3rd instar	30	6	rich_density_design	Type II-like	strong	0.654	0	0.0016		Boundary estimate	NA	181.3	HollingII
E. furcellata	adult	4th instar	30	6	rich_density_design	Type II-like	strong	0.737	0.0005	0.0014		Boundary estimate	NA	183.9	Rogers
E. furcellata	adult	Cocooned pupa	30	6	rich_density_design	Type II-like	strong	0.532	0	0.002		Boundary estimate	NA	169.4	HollingII
E. furcellata	adult	Naked pupa	30	6	rich_density_design	Type II-like	moderate	0.454	0	0.0039		Boundary estimate	NA	190.3	HollingII
H. patellifera	1st instar	4th instar	15	3	sparse_density_design	Type II-like	moderate	1.078	0.0136	0.0029	[0.0071, 0.0197]	Weakly identified	NA	47.16	HollingII
H. patellifera	1st instar	5th instar	15	3	sparse_density_design	Type II-like	moderate	1.427	0.0301	0.0037	[0.0218, 0.038]	Weakly identified	NA	51.68	HollingII
H. patellifera	2nd instar	4th instar	15	3	sparse_density_design	Type II-like	weak	0.875	0.0066	0.0041		Weakly identified	NA	53.11	HollingII
H. patellifera	2nd instar	5th instar	15	3	sparse_density_design	Type II-like	moderate	1.094	0.0069	0.0014	[0.0038, 0.01]	Weakly identified	NA	33.58	Rogers
H. patellifera	3rd instar	4th instar	15	3	sparse_density_design	ambiguous	weak	0.549	0	0.0124		Boundary estimate	NA	64.52	HollingII

H. patellifera	3rd instar	5th instar	15	3	sparse_density_design	Type II-like	moderate	1.669	0.0315	0.0051	[0.0211, 0.0415]	Weakly identified	NA	64.63	Rogers
Paratenodera sinensis Saussure	1st instar	4th instar	15	3	sparse_density_design	Type III-like	weak	1.064	0.0128	0.0064		Weakly identified	NA	70.78	Rogers
Paratenodera sinensis Saussure	1st instar	5th instar	15	3	sparse_density_design	Type II-like	weak	0.858	0.0025	0.0039		Boundary estimate	NA	54.02	HollingII
Paratenodera sinensis Saussure	2nd instar	4th instar	15	3	sparse_density_design	Type III-like	weak	1.143	0.0122	0.0025	[0.0068, 0.0173]	Weakly identified	NA	46.86	Rogers
Paratenodera sinensis Saussure	2nd instar	5th instar	15	3	sparse_density_design	Type III-like	weak	1.442	0.0285	0.0072	[0.0132, 0.0426]	Weakly identified	NA	72.95	Rogers
Paratenodera sinensis Saussure	3rd instar	4th instar	15	3	sparse_density_design	Type II-like	moderate	0.837	0.0304	0.009	[0.0101, 0.0487]	Weakly identified	NA	57.49	Rogers
Paratenodera sinensis Saussure	3rd instar	5th instar	15	3	sparse_density_design	ambiguous	weak	0.936	0	0.0023		Boundary estimate	NA	46.05	HollingII

Notes: Response form was classified as Type II-like, Type III-like, or ambiguous according to the procedure described in the Methods. *Th* identifiability was evaluated as stable, weakly identified, boundary estimate, or non-identifiable. *1/Th* is reported only when maximum predation capacity was considered sufficiently reliable for direct reporting. *AIC*, Akaike information criterion.
